# Bioengineering lungs: An overview of current methods, requirements, and challenges for constructing scaffolds

**DOI:** 10.3389/fbioe.2022.1011800

**Published:** 2022-10-28

**Authors:** Shahad Shakir, Tillie Louise Hackett, Leila B. Mostaço-Guidolin

**Affiliations:** ^1^ Department of Mechanical and Aerospace Engineering, Carleton University, Ottawa, ON, Canada; ^2^ Department of Anesthesiology, Pharmacology and Therapeutics, University of British Columbia, Vancouver, BC, Canada; ^3^ Centre for Heart Lung Innovation, University of British Columbia, Vancouver, BC, Canada; ^4^ Department of Systems and Computer Engineering, Carleton University, Ottawa, ON, Canada

**Keywords:** bioengineered lungs, scaffolds, recellularization, acellular lung scaffolds, artificial lung scaffolds, bioreactors, 3D bioprinting, electrospinning

## Abstract

Chronic respiratory diseases remain a significant health burden worldwide. The only option for individuals with end-stage lung failure remains Lung Transplantation. However, suitable organ donor shortages and immune rejection following transplantation remain a challenge. Since alternative options are urgently required to increase tissue availability for lung transplantation, researchers have been exploring lung bioengineering extensively, to generate functional, transplantable organs and tissue. Additionally, the development of physiologically-relevant artificial tissue models for testing novel therapies also represents an important step toward finding a definite clinical solution for different chronic respiratory diseases. This mini-review aims to highlight some of the most common methodologies used in bioengineering lung scaffolds, as well as the benefits and disadvantages associated with each method in conjunction with the current areas of research devoted to solving some of these challenges in the area of lung bioengineering.

## 1 Introduction

Chronic respiratory diseases including chronic obstructive pulmonary disease (COPD), asthma, and lung cancer combined are the third leading cause of death worldwide (De Santis, 2021). Over, four million people die prematurely from lung diseases per year, and they are predicted to continue to increase over the upcoming years (GBD 2015 Mortality and Causes of Death Collaborators, 2016). Currently, there is no cure for these diseases and lung transplantation (LTX) remains the only option for individuals with end-stage lung failure. Despite the improvement in organ preservation techniques, and specifically *ex vivo* lung perfusion (EVLP), alternative options are urgently required to increase available tissues for transplantation and close the gap in this clinical need (Dorrello et al., 2017; Chan et al., 2020). A novel research area that can assist with addressing this issue is bioengineered lung tissues. Although it remains a challenge to achieve a fully functional bioengineered lung that could be transplanted, new techniques are being explored to fabricate functioning *ex vivo* engineered lung tissues with proper gas exchange properties. These engineered models can be used as tools to aid in the development of new therapeutics and to understand lung physiology, cell-cell and cell-matrix interactions in disease models, and eventually increase tissue availability for lung transplantation (Gilpin et al., 2018).

**FIGURE 1:**
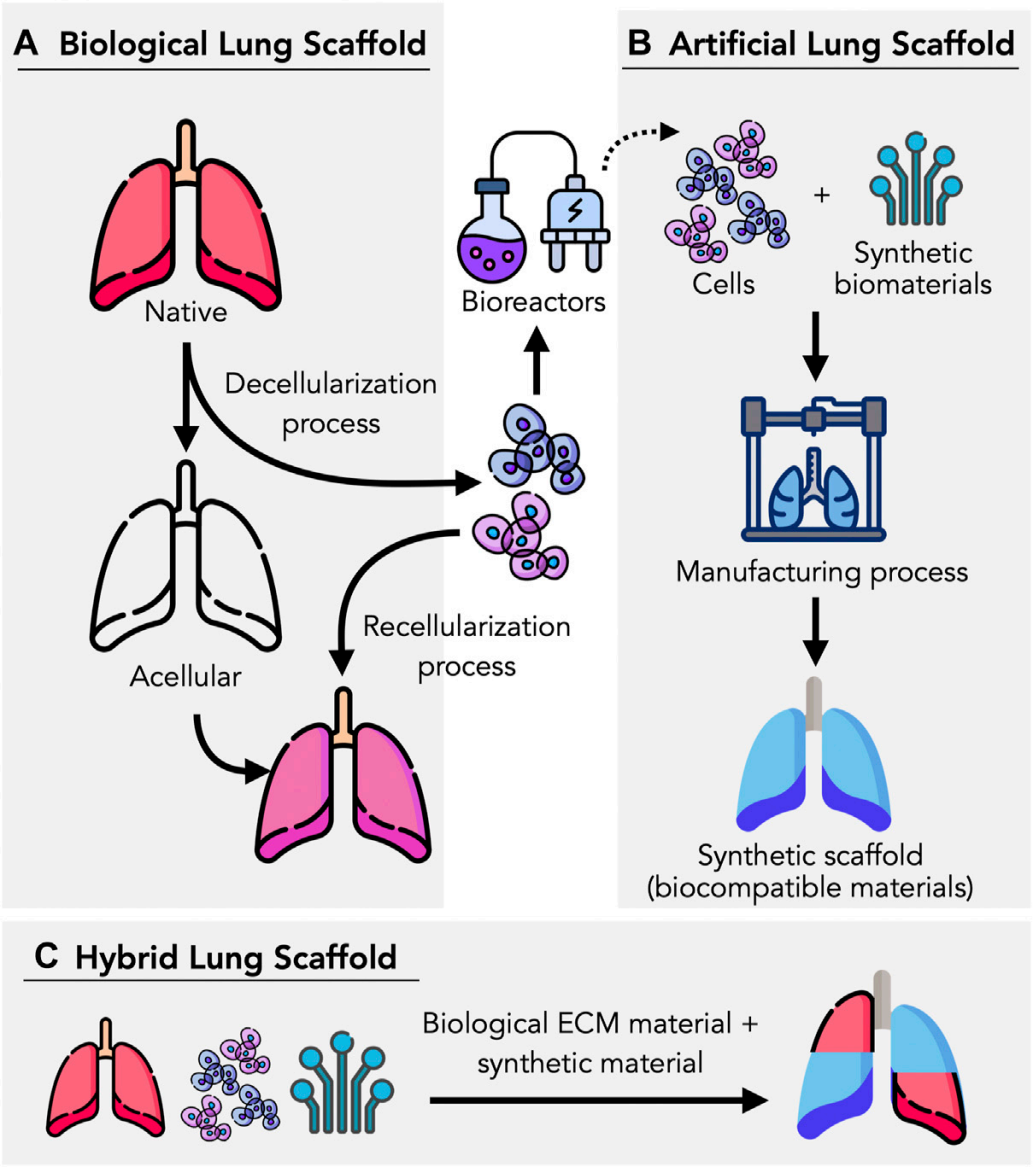
Lung tissue scaffolds can be bioengineering in different ways. **(A)** Biological lung scaffolds can be obtained from humans and animals, where they are decellularized using enzymatic agents and detergents to remove cellular components and achieve an ECM-based, acellular scaffold. **(B)** Artificial scaffolds can be manufactured based on a variety of methods (e.g., 3D bioprinting, electrospinning), utilizing synthetic biomaterials, cells, and bioinks. **(C)** By combining biological components such as cells and ECMs, hybrid scaffolds can be manufactured in combination with biocompatible materials which provide the necessary structure and mechanical proprieties to promote cell proliferation, adhesion and viability. Figure designed by Freepik with elements from flaticon.com.

The intricate native 3D architecture of the lung must be maintained, thus decellularized biological scaffolds from animal or human organs have become extremely useful templates for lung engineering (Wanczyk et al., 2021). The use of scaffolds eliminates the need to reconstruct the highly complex extracellular matrix (ECM) within the airways, parenchyma and vascular networks, offering an ideal acellular structure for seeding and recellularization. However, it is unknown to what extent the tissue and ECM components are preserved during decellularization, which is vital for the functionality of the bioengineered lung (De Santis et al., 2018; De Santis, 2021). Alternatively, synthetic lung scaffolds are another option to acellular scaffolds, where both synthetic and natural polymers can be used in the manufacturing process. Several approaches have been studied in manufacturing synthetic lungs such as 3D bioprinting, cryogelation, solvent-casting, and particulate-leaching techniques (De Santis et al., 2018; Eltom et al., 2019). In this mini-review, we aim to discuss recent advances and emerging technologies used in lung scaffolds bioengineering, as summarized in Figure 1. We also discuss some of the major requirements and challenges for future advancements.

## 2 Synthesizing biological (acellular) lung scaffolds

The limited accessibility of lung tissues available for LTX remains a major issue, and bioengineered tissues have the potential to bridge this gap. Bioengineered tissues rely on the incorporation of cells with biodegradable and biocompatible structures, known as scaffolds. Scaffolds mimic the extracellular matrix (ECM), by providing structural support, biological, chemical, and mechanical cues that influence the development of new tissues. Scaffolds act as a template to permit the transport of liquids, nutrients, gases, promote cell-cell interactions and ECM deposition, while reducing the risk of immunogenicity and proinflammatory responses through biocompatible and biodegradable properties (Prakash et al., 2015; Nikolova 2019; Nikolova and Chavali, 2019).

Despite the fact that lung tissue engineering efforts have typically trailed behind those of other organs, significant new developments using natural and synthetic scaffolds have been made. It is essential to investigate the characteristics necessary while choosing a scaffold to engineer a tissue. Properties such as strength, elasticity, nutrient transfer, cellular remodeling, and geometry should allow for proper tissue functionality (Nichols et al., 2013). Current approaches in the pre-clinical phase involve the use of decellularized biological and artificial lung scaffolds, seeded with cells sourced from the transplant recipient to promote tissue regeneration (Prakash et al., 2015).

### 2.1 Recapitulating biological lung scaffolds

The lung is a complex organ containing at least 40 distinct types of cells. These cells interact with an extracellular matrix (ECM), which is made up of different regional combinations of ECM proteins and glycosaminoglycans (e.g., proteoglycans and hyaluronan) that act as a scaffold to not only provide structure, but also help direct repair and regeneration after injury (Franks et al., 2008; Burgstaller et al., 2017). The use of biological lung scaffolds helps in retaining the complex ECM and scaffold structure, making this method a promising technique for lung bioengineering. Researchers have been exploring transplantation in animal models, and successful transplantation using acellular scaffolds in rodent and porcine models has been achieved (Tsuchiya et al., 2014; Wanczyk et al., 2021). To use these acellular scaffolds, a decellularization process that removes cells and cellular materials from the tissue or organ is required. Decellularization techniques leave an intact 3D scaffold and ECM composition, which can later be recellularized with necessary cells to carry proper functions (Gilpin and Yang, 2017; Mendibil et al., 2020; Wanczyk et al., 2021). Sections below will dive deeper into decellularization and recellularization techniques.

#### 2.1.1 Decellularization techniques

The lung decellularization process is carried out using chemical and physical methods, or a combination of both. Chemical methods include using genzymatic agents, and detergents. Ionic and nonionic detergent-based solutions such as sodium dodecyl sulfate (SDS), sodium deoxycholate (SDC), and Triton X-100, are the most used to wash away cell debris (Wallis et al., 2012; Lin et al., 2013), but each method possesses side effects. SDS is cytotoxic, and it requires an extensive washing process, it can also denaturalize proteins, and the residual matrix may contain nuclear and cytoplasmic waste (Gilpin and Yang, 2017; Mendibil et al., 2020). SDC can cause aggregation of DNA when used without DNase, however, it is a simple, versatile and nondisruptive method for tissue decellularization (Meezan et al., 1975; Gilpin and Yang, 2017). Triton X-100 is not recommended for decellularization of ECM, where lipids and glycosaminoglycans (GAGs) are important for cell specific functions, but it is less damaging to the structure of tissues than ionic surfactants (Gilpin and Yang, 2017; Mendibil et al., 2020).

Alternatively, physical methods include the use of osmotic shock, sonication, and freezing-thawing to break the cell membrane (Mendibil et al., 2020; Rabbani et al., 2021). Hypotonic and hypertonic solutions are used to deliver an osmotic shock kills cells through cell explosion, and it releases the cell waste to the matrix. It is important to note that the cell waste released should be managed and is considered in the design of the decellularization procedure (Reing et al., 2010; Mendibil et al., 2020). The sonication and agitation methods are commonly used to facilitate chemical agent infiltration and to induce cell lysis, however, the entire process can induce significant damage to the ECM. Lastly, freezing methods utilize crystals that are created in the freezing process, which are employed to damage cell membranes. The advantage of this method includes maintaining the ECM proteins, and mechanical properties, but it is unknown what degree the ECM protein structure is compromised (Gilpin and Yang, 2017; Mendibil et al., 2020).

These methods are known to retain most of the macro and microstructure of the lung and leave all ECM components intact. By unaffecting the ECM structure, it is possible to aid the promotion of cell proliferation after recellularization, as it contains proteins and biochemical cues that would direct cells and facilitate cell communication (De Santis et al., 2018; Young 2019). However, it is not known to what degree the microarchitectural, airway and vascular systems, and ECM components (such as collagen, elastin, fibronectin, and GAGS) have been preserved, leading to possible tissue functionality limitations (Balestrini, Niklason, 2015).

The mechanical properties and biomimicry of hydrogels are crucial to promote tissue growth. Lung tissues enzymatically solubilized have been shown to be a promising method to obtain dECM (decellularized ECM) to coat transwell inserts to enhance cell adhesion and growth (Barreiro Carpio et al., 2021). This work provided a complete investigation of how different dECM solutions affects cell viability and proliferation on hydrogels.

A recent study by *Skolasinski and Panoskaltsis-Mortari* suggests leaving the vasculature intact while only decellularizing the airway epithelium can benefit in preserving some architecture and ECM components (Skolasinski and Panoskaltsis-Mortari, 2018; Wanczyk et al., 2021). Additionally, Obata et al. used natural soap, potassium laurate, as a decellularization detergent that was found to be less abrasive and showed to be effective in removing cellular components and cells while preserving the 3D lung architecture better (Obata et al., 2019). Lastly, to promote cellular adhesion and differentiation, scaffolds can be replenished with ECM proteins and coated with agents that improve vascularization in organs (Shojaie et al., 2015; Young et al., 2019; Yuan et al., 2019; Uhl et al., 2020; Wanczyk et al., 2021).

It is important to also highlight that the procedure does not consider cytocompatibility related to residual cell debris and unremoved decellularization agents in the tissue (Gilpin and Wagner, 2018). Lastly, an ideal decellularization protocol has not been found yet, wherein published literature, different studies state different administrative ways, solution volumes, detergent concentration, and length of exposure. This may influence the recellularization process and the eventual bioengineered tissue (Balestrini et al., 2015; Colvin and Yeage, 2015) Therefore, more research is required to standardize the current decellularization process Figure 1.

#### 2.1.2 Recellularization process: An overview

The recellularization process involves repopulating cells into the acellular scaffold, to promote cell attachments, proliferation, and migration that would allow for organ regeneration (Ahmed et al., 2021) Autologous cells derived from the patient are considered an ideal source as they can reduce adverse immunological response and the need for long-term immunosuppressive medications (Hillebrandt et al., 2019). Common cell types that have been successfully used in the recellularization of acellular lung scaffolds include induced pluripotent stem cells (iPSCs), embryonic stem cells (ESCs), and lung-derived progenitor cells (Wagner et al., 2013; Weiss et al., 2013; Tsuchiya et al., 2014; Scarritt et al., 2015). iPSCs cells that are derived from reprogrammed adult somatic cells can be differentiated into the epithelial cells, making them an ideal seeding source and show promise in the development of lung-specific cells (Prakash et al., 2015; Aboul-Soud et al., 2021). While candidates for stem/progenitor cells include alveolar progenitor cells (AEPCs) that are isolated from human lungs, they require certain cues that are secreted by the ECM, which emphasizes the need for a decellularization protocol that retains important ECM components (Tsuchiya et al., 2014; Prakash et al., 2015).

Although multiple studies show the potential of these different cell sources to recellularize a lung scaffold, the challenge remains in choosing appropriate cell types, quantities, and combinations to achieve full tissue functionality (Echeverria Molina et al., 2021). Emerging data obtained from advanced single-cell analyses and multi-omics approaches, combined with 3D image analyses can enhance the biofabrication of functional lung tissues, where data regarding cell identity, localization, and abundance can be evaluated. Furthermore, trajectory inference methodologies can be used to infer the sequence of lung cells, allowing to engineer mature lung types with greater capabilities to restore tissue structure and functionality (Jackson et al., 2020; Wanczyk et al., 2021).

It is important to note that when considering the sources of the biological scaffold, the donor tissue does not have to be of human origin, and tissues can be sources from similar species such as porcine and non-human primates (De Santis et al., 2018; Ribitsch et al., 2020). Using non-human scaffolds provides a uniform scaffold for lung bioengineering, which reduces some of the limitations that human lungs possess, however, ethical and health issues with animal scaffolds remain a problem (Nichols et al., 2013). Although acellular biological lungs show great potential, this approach is challenging due to the shortage of suitable human lungs available, as well as the potential heterogeneity and xenogeneity issues, making an alternative method extremely needed (Mohgan et al., 2022, De Santiset al., 2018).

## 3 Artificial lung scaffolds

Despite the advances that have been made regarding using biological (acellular) lung scaffolds, their heterogeneity and potential xenogeneic concerns make this technique difficult to scale-up in a repeatable and regulated manner (Chan and Leong, 2008). Artificial, or synthetic, lung scaffolds can be utilized instead of acellular scaffolds, where both synthetic and natural polymers are typically used in the manufacturing. One advantage of using synthetic materials to biofabricate scaffolds is the ability to tailor their biological and physical properties to achieve a desirable scaffold (Nikolova and Chavali, 2019; Matai et al., 2020). Although researchers have explored using methods such as 3D bioprinting, cryogelation, solvent-casting, and particulate-leaching techniques to engineer tissues, challenges are still present when the goal is to generate functional lung tissues (De Santis et al., 2018; Eltom et al., 2019; Xie et al., 2020). Limitations include the lung’s complex airway branching structure, and the challenge of low biocompatibility and hemocompatibility, which are the ability of being compatible with a living tissue and with blood, respectively (Tebyanian et al., 2019). To date, many techniques have been investigated to artificially engineer separately tracheal, bronchial, and parenchymal lung tissues. Specifically, tracheal lung tissue engineering has been studied more extensively due to the trachea’s architecture and tubular structure (Jungebluth et al., 2011; Chang et al., 2014; Jungebluth et al., 2014; Crowley et al., 2015; De Santis et al., 2018).

Promising prototypes show that epithelialisation and vascularisation of the grafts can be achieved through different procedures. On the other hand, there is a lack of research surrounding 3D-bioprinting and organoids techniques for parenchymal lung tissues. Techniques such as foaming, porogen-solvent, and self-assembly of microspheres show great promise, but the fabricated scaffold lacks the vasculature and gas exchanges characteristics required for functional lung tissue (Andrade et al., 2007; Singh et al., 2013; Ling et al., 2014; Wilkinson et al., 2017; De Santis et al., 2018). Below we discuss the various techniques that have been used to develop artificial lung scaffolds Table 1.

**TABLE 1:** Summary of lungs bioengineering emerging concepts and procedures, and their main applications and limitations.

	Applications	Limitations	Selected references
Biological Lung Scaffolds			
Overview	■ Offers an ideal acellular structure for seeding and recellularization.	■ Donor shortages.	Tsuchiya et al. (2014), Wanczyk et al. (2021), Chan and Leong, (2008).
	■ Aid in retaining the complex biological ECM and scaffold structure	■ Difficult to scale up in a repeatable and regulated manner due	
		■ Many donated lungs do not meet the criteria for transplantation	
Decellularization	■ Removes cells and cellular materials from the tissue or organ	■ Not known to what degree is macro and microstructure is preserved	De Santis et al. (2018), Young (2019), Barreiro Carpio et al. (2021), Balestrini and Niklason, (2015), Gilpin and Wagner, (2018), Colvin and Yeager, (2015), Balestrini et al. (2015).
	■ Retain most of the macro and microstructure of the lung	■ Procedure does not consider cytocompatibility related to cell debris and unremoved decellularization agents	
	■ Leave ECM intact, improves cell proliferation and recellularization	■ No standard decellularization protocol available yet	
Recellularization	■ Repopulating scaffolds with cells, to promote cell attachment, proliferation, and migration to carry proper functions and organ regeneration	■ Challenge remains in choosing appropriate cell types, quantities, and combinations.	Ahmed et al. (2021), Echeverria Molina et al. (2021), Tsuchiya et al. (2014), Prakash et al. (2015)
		■ No standard recellularization protocol available yet	
Artificial Lung Scaffolds			
Overview	■ Helps with organ shortages issues.	■ Lung’s complex hierarchical structure makes it difficult to biofabricate a scaffold.	Nikolova and Chavali, (2019), Matai et al. (2020), Xie et al. (2020), Eltom et al. (2019), De Santis et al. (2018), Tebyanian et al. (2019), Ling et al. (2014), Andrade et al. (2007), Singh et al. (2013), Wilkinson et al. (2017)
	■ Possible to tailor their biological and physical properties to achieve a desirable scaffold.	■ Scaffold lack the vasculature and gas exchanges characteristics required for a functional lung tissue.	
3D Bioprinting	■ Allow for custom-made 3D designs.	■ Challenge in choosing appropriate manufacturing methods, that allow bioprinting at a microscale resolution to achieve key features of lung tissues.	Rider et al. (2018), Mahfouzi et al. (2021a), Xie et al. (2020), Barreiro Carpio et al. (2021), Frejo, Grande, (2019), Galliger et al. (2019), Grigoryan et al. (2019), Suki and Bates, (2008), Ozbolat and Yu, (2013), De Santis et al. (2021).
	■ Scaffolds can be bioprinted with growth factors or drugs that can be released, expediting tissue formation.	■ The lack of an appropriate commercial bioink that mimics the true ECM-like elements.	
Electrospinning	■ Electrospun scaffolds can be fabricated to any size, and fiber parameters	■ Challenges in controlling geometry of the scaffolds, damage to encapsulated cells, and insufficient control of cell patterning.	Jun et al. (2018), De Santis et al. (2018), Young et al. (2017), Asadian et al. (2020), Abbasi et al. (2014), Owida et al. (2022), Persano et al. (2013).
	■ Capable of allowing ideal cell interactions, adherence, proliferation, migration, and differentiation with an appropriate plasma treatment	■ Further properties such as mechanical strength and potential toxicity need to be further studied	
	■ Simple, low-cost, and is an adaptable technology		
Bioreactors	■ Provide controlled and prespecified environmental settings, allowing the needed biological processes to occur to support cells	■ Achieving a uniform temperature across the chamber	Farré et al. (2018), Mahfouzi et al. (2021b), García-Gareta et al. (2020).
	■ Expose re-seeded scaffolds to physical and biochemical stimuli	■ Achieving a sterile environment	
		■ Achieving proper pH control and waste removal	

### 3.1 Potential manufacturing methods to generate artificial scaffolds for lung tissue engineering

Different manufacturing methods have been researched for manufacturing porous structures for tissue engineering. Methods such as electrospinning, 3D bioprinting, cryogelation, solvent-casting, and particulate-leaching techniques have been used (De Santis et al., 2018; Eltom et al., 2019; Xie et al., 2020). Particularly in terms of lungs, electrospinning and 3D bioprinting have been gaining more attention recently. Electrospinning has emerged as a promising method and is considered to be effective in producing porous structures composed of thin, nano-fibers, that are capable of providing enough support for the cell attachments, proliferation, and differentiation (Persano et al., 2013; Owida et al., 2022). However, challenges such as geometry control, cell damage, and insufficient control of cell patterning remain an issue (Jun et al., 2018).

On the other hand, 3D Bioprinting has emerged as a promising source for bioengineering tissues. Using 3D bioprinting allows researchers to create tissues mimicking natural tissues, that can aid in studying *in-vitro* models and eventually be used in tissue transplantation (Kolesky et al., 2016; Feinberg and Miller, 2017; Zhu et al., 2017). Despite its great promise, 3D printing a fully functional lung structures remain a challenge and is beyond current capabilities. Research is also currently limited in regard to attempts involving 3D bioprinting of lung tissues (Barreiro Carpio et al., 2021). Below, these manufacturing methods are explained in more depth.

#### 3.1.1 Electrospinning

Electrospinning has emerged as one of the methods used to produce fibrillar scaffolds and has been extensively used to engineer the trachea scaffolds (Young et al., 2017; De Santis et al., 2018). Electrospun scaffolds can be fabricated to any size, and fiber parameters such as density, composition, and orientation can be manipulated to create a reliable ECM network (Jun et al., 2018; De Santis et al., 2018; Young et al., 2017).

It has been shown that these fabricated lung scaffolds have the ability to provide ECM fiber orientation and mechanical properties comparable to native lungs (Young et al., 2017; Nguyen-Truong et al., 2020). Electrospinning techniques are also capable of allowing ideal cell interactions and adherence, as well as cell proliferation, migration, and differentiation with an appropriate plasma treatment (Abbasi et al., 2014; Asadian et al., 2020). Moreover, to encourage successful tissue transplantation, scaffolds can incorporate growth factors or drugs that can be released, expediting tissue formation, and allowing the transplanted tissue to achieve homeostasis (Howard et al., 2008). There are many advantages of using electrospinning to create artificial scaffolds. Electrospinning is simple, low-cost, and is an adaptable technology with a great potential for developing multifunctional materials for application in tissue engineering. It has also been shown to be a valuable method to create ECM-mimicking structures, making scaffolds have identical ECM structures to those of native tissues than other conventional methods (Persano et al., 2013; Owida et al., 2022).

Although electrospinning has emerged as a favourable method used in multiple fields of tissue engineering, and shows a promising technique in artificial lung fabrication, challenges to control the geometry of the fabricated organ, damage to encapsulated cells, and insufficient control of cell patterning remains an issue, since cells must remain viable, and the constructs must preserve their structural integrity during/post printing process (Jun et al., 2018). Additionally, the vast majority of published research has been done *in vitro*. As a result, the composition and structure must be adjusted for *in vivo* applications in future research. Lastly, for ultimate clinical application, properties such as mechanical strength, cell infiltration, impediment, and potential toxicity of electrospun scaffolds need to be further studied (Owida et al., 2022).

#### 3.1.2 3D-bioprinting

Amongst the most versatile engineering approaches that allow for custom-made 3D designs in tissue engineering is 3D-bioprinting, which has emerged as a powerful technique in producing synthetic scaffolds for organs (Rider et al., 2018). 3D bioprinting has been an attractive tool that is being explored to engineer organs with the ultimate goal of producing transplantable tissues, and in the case of the lung, for creating artificial lung scaffolds (Mahfouzi et al., 2021a). Significant progress has been made that allows for the 3D bioprinting of the trachea and bronchus, but the challenge remains in choosing appropriate manufacturing methods, that allow bioprinting at a microscale resolution to achieve key features of lung tissues (Frejo and Grande, 2019; Galliger et al., 2019; Xie et al., 2020). Additionally, scaffolds design criteria need to also be addressed to achieve functional tissues. The final structure must mimic that of a biological lung and should meet the geometrical and characteristics requirements. Appropriate viscoelastic and mechanical traits must allow for lung inflation, deflation, and normal blood flow (Suki and Bates, 2008; Grigoryan et al., 2019; Mahfouzi et al., 2021b). Most importantly, the thin air blood barrier must be properly fabricated, to allow for efficient gas exchange (Horváth et al., 2015).

Speed, precision, and resolution parameters are key factors in choosing the bioprinting methods (e.g. extrusion, microfluidics, inkjet, laser-assisted) (Ozbolat and Yu, 2013; Murphy and Atala, 2014; Mandrycky et al., 2016; Mahfouzi et al., 2021a). In regard to the bioprinting of lung parenchyma, stereolithography has been used to provide highly precise construct with high resolution. For the bioprinting of tracheal structure, however, extrusion bioprinters were found to be a better option since they can support high cell densities, and different biopinks and biomaterials (Mahfouzi et al., 2021b).

Additionally, selecting a proper bioink is a critical step in 3D bioprinting. Bioinks that provide proper properties related to mechanical strength, flexibility, biocompatibility, biodegradability, bioabsorbability, and printability are essential to achieve a functioning organ (Mahfouzi et al., 2021a). They should also have properties needed to allow for specific biological cues that guide cell growth, differentiation, and migration (Mahfouzi et al., 2021b; Doyle and O’grady, 2013). One of the limiting factors of 3D bioprinting lung scaffolds is the lack of an appropriate commercial bioink that mimics the true ECM-like elements (De Santis et al., 2021) However, recent research by De Santis et al. showed a promising proof-of-concept for bioprinting human airways using a hybrid bioink, which is composed of alginate and a natural polymer, and reinforced with ECM from decellularized tissues (rECM) (De Santis et al., 2021). It can maintain biological properties while supporting tissue growth, as well as provide bioprinted constructs that are proangiogenic, biocompatible, and support new blood vessel formation (De Santis et al., 2021)

Recreating the human lung architecture is a challenging task with a high level of difficulty. Although current technologies are still unable to achieve the level or resolution needed for a functional, 3D bioprinted lung, due to its complex vasculature and architecture, it is important to highlight key challenges that need to be addressed to achieve a transplantable organ (Barreiro Carpio et al., 2021). The alveolar epithelium and vascularization of the lung needs to be reproduced accurately, requiring a specific spatial resolution that currently is a challenge for existing bioprinters (Weibel, 2015). To add, the bronchi, bronchiole, and alveoli not only present geometrical challenges for current bioprinters, but also require cells to be deposited at certain locations to form a functional membrane. Lastly, the limited biomaterials available for the printing process present a great level of difficulty to mimic the physical properties (*e.g.,* stiffness, elasticity, and gap permeability) of the lung, which are vital to its function during the process of breathing (Barreiro Carpio et al., 2021).

### 3.2 Bioreactors for cell expansion, differentiation, decellularization and re-cellularization

Bioreactors are critical in the bioengineering of lungs. Due to the unique lung anatomy and physiology, the use of bioreactors is crucial to biofabricate lung scaffolds, both artificial and biological. They provide controlled and prespecified environmental settings, allowing the biological processes needed to occur to support cell growth and differentiation, tissue decellularization and recellularization, and monitoring cell cultures (Farré et al., 2018; Mahfouzi et al., 2021a). They expose re-seeded scaffolds to physical and biochemical stimuli, allowing cells to undergo differentiation and regeneration within developing scaffolds. These stimuli can influence cell seeding, nutrient uptake in the medium, and mechanical forces (García-Gareta et al., 2020). Several bioreactors types exist for different applications.

Some examples include bioreactor systems for decellularization of scaffolds by perfusion and recellularization by perfusion and ventilation (Talò et al., 2020; Mahfouzi et al., 2021b). Others are systems used for large-scale cell cultures, which are composed of rotating bioreactors that differentiate epithelium cells for lung bioengineering by exposing cells to air and liquid (Ghaedi et al., 2014; Mahfouzi et al., 2021a). However, in regard to bioreactors involved in re- and decellularization of lungs, further limitations need to be addressed, such as achieving a uniform temperature across the chamber, sterile environment, pH control, and proper waste removal (Mahfouzi et al., 2021b). Lastly, ensuring adequate nutrient and oxygen supply available for the tissue is crucial. In the case of lung bioreactors, the organ should be exposed to controlled perfusion, ventilation, and gas exchange (Farré et al., 2018).

## 4 Discussion and future perspectives

Lung bioengineering requires a delicate balance between choosing the suitable scaffold material and enabling ECM function. Advantages and limitations of the different methods are summarized in Table 1. The above mentioned techniques described in this review offer a wealth of opportunities in lung bioengineering, and will lead to advancement for future transplanation. However, substantial experimental work is still needed to translate this technology into clinical practice. Ethical and legality issues need to be addressed before starting trials in humans, which include implementing strict regulations regarding the starting biomaterials (cells and scaffolds), methodology, and reliability and availability of materials and processes used (Farré et al., 2018). In the pre-clinical stages, many ethical considerations must be considered, including appropriate reporting, distribution of results, data integrity, and ensuring that every study done is planned to generate results suitable for deciding on the next research steps. Further, challenges such as safety and efficacy, scaling the bioengineered lungs to fit the patient’s body, choosing how biological materials are regulated and distributed, and cost of manufacturing need to be addressed before moving into the pre-clinical trials phase (Farré et al., 2018; De Santis et al., 2021).

To move forward with creating transplantable tissues, procedures regarding decellularization and recellularization must be established, and a proper framework that is sufficient to fully elucidate the physiological and biochemical interactions must be developed. Additionally, prior to clinical trials, the short and long outcomes of these transplantable tissues must be studied in animal models. This will aid in establishing regulatory frameworks and good manufacturing practice (GMP) standards regarding patient safety (Taylor et al., 2014). To date, the criteria used to evaluate bioengineered lung tissue prior to clinical trials remain undefined, although using measures similar to those that are used in EVLP may be reasonable as a first step. It is vital that researchers, physicians, and regulatory authorities collaborate to build these new frameworks ((Tsuchiya et al., 2014; Elliott et al., 2017; Mohgan et al., 2022)

In contrast, the use of acellular lung scaffolds is highly dependent on the donor material. Often, many donated lungs do not meet clinical criteria for transplantation, especially when the lungs are donated after cardiac death. Comorbidities such as chronic obstructive pulmonary disease (COPD), asthma) and immunogenicity are also important factors (Tsuchiya et al., 2014). Future research can explore ways to improve the approaches used in donated lungs after cardiac death, which may include using bioengineering methods to generate a reproduceable functional lung tissue (Chan et al., 2020).

In conclusion, the research that has been made in lung bioengineering has been used to better understand the biological process of lung repair in during injury and has helped replace the need for animals to model disease (De Santis et al., 2021). The potential rewards of success in developing lung scaffolds holds great promise for patients suffering from multiple chronic pulmonary diseases. However, it clearly remains an ambitious goal to be continually tackled by multidisciplinary collaborations and team efforts amongst bioengineers, physiologists, and clinicians. Advances related to scaffold design and production, biocompatibility of materials, and the ability to maintain appropriate biomechanical properties, are only a few challenges behind the development of lung scaffolds. The development of mathematical models and techniques to improve cell viability, proliferation, adhesion at the same time once can control ECM properties, will allow the discoveries to provide stepping stones toward the development of clinically useful tissue-engineered lung.

## References

[B1] Abbasi N. Soudi S. Hayati-Roodbari N. Dodel M. Soleimani M. (2014). The effects of plasma treated electrospun nanofibrous poly (ε-caprolactone) scaffolds with different orientations on mouse embryonic stem cell proliferation. Cell. J. 16 (3), 245–254. 24611137 PMC4204185

[B2] Aboul-Soud M. A. M. Alzahrani A. J. Mahmoud A. (2021). Induced pluripotent stem cells (iPSCs)—roles in regenerative therapies, disease modelling and drug screening. Cells 10, 2319. 10.3390/cells10092319 34571968 PMC8467501

[B3] Ahmed E. Saleh T. Xu M. (2021). Recellularization of native tissue derived acellular scaffolds with mesenchymal stem cells. Cells 10 (7), 1787. 10.3390/cells10071787 34359955 PMC8304639

[B4] Andrade C. F. Wong A. P. Waddell T. K. Keshavjee S. Liu M. (2007). Cell-based tissue engineering for lung regeneration. Am. J. Physiology-Lung Cell. Mol. Physiology 292 (2), L510–L518. 10.1152/ajplung.00175.2006 17028264

[B5] Asadian M. Chan K. V. Norouzi M. Grande S. Cools P. Morent R. (2020). Fabrication and plasma modification of nanofibrous tissue engineering scaffolds. Nanomater. (Basel, Switz. 10 (1), 119. 10.3390/nano10010119 PMC7023287 31936372

[B6] Balestrini J. L. Gard A. L. Liu A. Leiby K. L. Schwan J. Kunkemoeller B. (2015). Production of decellularized porcine lung scaffolds for use in tissue engineering. Integr. Biol. quantitative Biosci. Nano macro 7 (12), 1598–1610. 10.1039/c5ib00063g PMC4666745 26426090

[B7] Balestrini J. L. Niklason L. E. (2015). Extracellular matrix as a driver for lung regeneration. Ann. Biomed. Eng. 43 (3), 568–576. 10.1007/s10439-014-1167-5 25344351 PMC4380778

[B9] Barreiro Carpio M. Dabaghi M. Ungureanu J. Kolb M. R. Hirota J. A. Moran-Mirabal J. M. (2021). 3D bioprinting strategies, challenges, and opportunities to model the lung tissue microenvironment and its function. Front. Bioeng. Biotechnol. 9, 773511. 10.3389/fbioe.2021.773511 34900964 PMC8653950

[B11] Burgstaller G. Oehrle B. Gerckens M. White E. S. Schiller H. B. Eickelberg O. (2017). The instructive extracellular matrix of the lung: Basic composition and alterations in chronic lung disease. Eur. Respir. J. 50, 1601805. 10.1183/13993003.01805-2016 28679607

[B12] Chan B. P. Leong K. W. (2008). Scaffolding in tissue engineering: General approaches and tissue-specific considerations. Eur. Spine J. 17 (4), 467–479. 10.1007/s00586-008-0745-3 19005702 PMC2587658

[B13] Chan P. G. Kumar A. Subramaniam K. Sanchez P. G. (2020). *Ex vivo* lung perfusion: A review of research and clinical practices. Semin. Cardiothorac. Vasc. Anesth. 24 (1), 34–44. 10.1177/1089253220905147 32036756

[B14] Chang J. W. Park S. A. Park J. K. Choi J. W. Kim Y. S. Shin Y. S. (2014). Tissue-engineered tracheal reconstruction using three-dimensionally printed artificial tracheal graft: Preliminary report. Artif. organs 38 (6), E95–E105. 10.1111/aor.12310 24750044

[B15] Colvin K. L. Yeager M. E. (2015). Applying biotechnology and bioengineering to pediatric lung disease: Emerging paradigms and platforms. Front. Pediatr. 3, 45. 10.3389/fped.2015.00045 26106589 PMC4460801

[B16] Crowley C. Birchall M. Seifalian A. M. (2015). Trachea transplantation: From laboratory to patient. J. Tissue Eng. Regen. Med. 9 (4), 357–367. 10.1002/term.1847 26052583

[B18] De Santis M. M. Alsafadi H. N. Tas S. Bölükbas D. A. Prithiviraj S. Da Silva I. (2021). Extracellular-matrix-reinforced bioinks for 3D bioprinting human tissue. Adv. Mater. Deerf. Beach, Fla.) 33 (3), e2005476. 10.1002/adma.202005476 PMC11469085 33300242

[B19] De Santis M. M. Bölükbas D. A. Lindstedt S. Wagner D. E. (2018). How to build a lung: Latest advances and emerging themes in lung bioengineering. Eur. Respir. J. 52 (1), 1601355. 10.1183/13993003.01355-2016 29903859

[B20] De Santis M. M. (2021). Next generation bioengineering of lung tissue for transplantation. Doctoral Dissertation (Lund: Lund University, Faculty of Medicine).

[B21] Dorrello N. V. Guenthart B. A. O'Neill J. D. Kim J. Cunningham K. Chen Y. W. (2017). Functional vascularized lung grafts for lung bioengineering. Sci. Adv. 3 (8), e1700521. 10.1126/sciadv.1700521 28875163 PMC5576878

[B22] Doyle D. J. O’grady K. F. (2013). Physics and modeling of the airway. Benumof Hagb. Airw. Manag. 2013, 92–117. 10.1016/b978-1-4377-2764-7.00004-x

[B24] Echeverria Molina M. I. Malollari K. G. Komvopoulos K. (2021). Design challenges in polymeric scaffolds for tissue engineering. Front. Bioeng. Biotechnol. 9, 617141. 10.3389/fbioe.2021.617141 34195178 PMC8236583

[B25] Elliott M. J. Butler C. R. Varanou-Jenkins A. Partington L. Carvalho C. Samuel E. (2017). Tracheal replacement therapy with a stem cell-seeded graft: Lessons from compassionate use application of a GMP-compliant tissue-engineered medicine. Stem Cells Transl. Med. 6 (6), 1458–1464. 10.1002/sctm.16-0443 28544662 PMC5689750

[B26] Eltom A. Zhong G. Muhammad A. (2019). Scaffold techniques and designs in tissue engineering functions and purposes: A review. Adv. Mater. Sci. Eng. 2019, 3429527. 10.1155/2019/3429527

[B27] Farré R. Otero J. Almendros I. Navajas D. (2018). Bioengineered lungs: A challenge and an opportunity. Arch. bronconeumologia 54 (1), 31–38. 10.1016/j.arbres.2017.09.002 29102342

[B28] Feinberg A. W. Miller J. S. (2017). Progress in three-dimensional bioprinting. MRS Bull. 42, 557–562. 10.1557/mrs.2017.166

[B29] Franks T. J. Colby T. V. Travis W. D. Tuder R. M. Reynolds H. Y. Brody A. R. (2008). Resident cellular components of the human lung: Current knowledge and goals for research on cell phenotyping and function. Proc. Am. Thorac. Soc. 5 (7), 763–766. 10.1513/pats.200803-025HR 18757314

[B30] Frejo L. Grande D. A. (2019). 3D-bioprinted tracheal reconstruction: An overview. Bioelectron. Med. 5, 15. 10.1186/s42234-019-0031-1 32232104 PMC7098220

[B31] Galliger Z. Vogt C. D. Panoskaltsis-Mortari A. (2019). 3D bioprinting for lungs and hollow organs. Transl. Res. 211, 19–34. 10.1016/j.trsl.2019.05.001 31150600 PMC6702089

[B32] García-Gareta E. Abduldaiem Y. Sawadkar P. Kyriakidis C. Lali F. Greco K. V. (2020). Decellularised scaffolds: Just a framework? Current knowledge and future directions. J. Tissue Eng. 11, 204173142094290. 10.1177/2041731420942903 PMC7376382 32742632

[B33] GBD 2015 Mortality and Causes of Death Collaborators (2016). Global, regional, and national life expectancy, all-cause mortality, and cause-specific mortality for 249 causes of death, 1980-2015: A systematic analysis for the global burden of disease study 2015. Lancet (London, Engl. 388 (10053), 1459–1544. 10.1016/S0140-6736(16)31012-1 PMC5388903 27733281

[B35] Ghaedi M. Mendez J. J. Bove P. F. Sivarapatna A. Raredon M. S. B. Niklason L. E. (2014). Alveolar epithelial differentiation of human induced pluripotent stem cells in a rotating bioreactor. Biomaterials 35, 699–710. 10.1016/j.biomaterials.2013.10.018 24144903 PMC3897000

[B36] Gilpin A. Yang Y. (2017). Decellularization strategies for regenerative medicine: From processing techniques to applications. BioMed Res. Int. 2017, 1–13. 10.1155/2017/9831534 PMC5429943 28540307

[B37] Gilpin S. E. Wagner D. E. (2018). Acellular human lung scaffolds to model lung disease and tissue regeneration. Eur. Respir. Rev. 27 (148), 180021. 10.1183/16000617.0021-2018 29875137 PMC9488127

[B39] Grigoryan B. Paulsen S. J. Corbett D. C. Sazer D. W. Fortin C. L. Zaita A. J. (2019). Multivascular networks and functional intravascular topologies within biocompatible hydrogels. Sci. (New York, N.Y.) 364 (6439), 458–464. 10.1126/science.aav9750 PMC7769170 31048486

[B40] Hillebrandt K. H. Everwien H. Haep N. Keshi E. Pratschke J. Sauer I. M. (2019). Strategies based on organ decellularization and recellularization. Transpl. Int. 32, 571–585. 10.1111/tri.13462 31099920

[B41] Horváth L. Umehara Y. Jud C. Blank F. Petri-Fink A. Rothen-Rutishauser B. (2015). Engineering an *in vitro* air-blood barrier by 3D bioprinting. Sci. Rep. 5, 7974. 10.1038/srep07974 25609567 PMC4303938

[B42] Howard D. Buttery L. D. Shakesheff K. M. Roberts S. J. (2008). Tissue engineering: Strategies, stem cells and scaffolds. J. Anat. 213 (1), 66–72. 10.1111/j.1469-7580.2008.00878.x 18422523 PMC2475566

[B45] Jackson H. W. Fischer J. R. Zanotelli V. Ali H. R. Mechera R. Soysal S. D. (2020). The single-cell pathology landscape of breast cancer. Nature 578 (7796), 615–620. 10.1038/s41586-019-1876-x 31959985

[B48] Jun I. Han H. S. Edwards J. R. Jeon H. (2018). Electrospun fibrous scaffolds for tissue engineering: Viewpoints on architecture and fabrication. Int. J. Mol. Sci. 19 (3), 745. 10.3390/ijms19030745 PMC5877606 29509688

[B49] Jungebluth P. Alici E. Baiguera S. Blomberg P. Bozóky B. Crowley C. (2011). Retracted: Tracheobronchial transplantation with a stem-cell-seeded bioartificial nanocomposite: A proof-of-concept study. Lancet 378 (9808), 1997–2004. (Retraction published Lancet. 2018 Jul 7;392(10141):11). 10.1016/S0140-6736(11)61715-7 22119609

[B50] Jungebluth P. Haag J. C. Sjöqvist S. Gustafsson Y. Beltrán Rodríguez A. Del Gaudio C. (2014). Tracheal tissue engineering in rats. Nat. Protoc. 9 (9), 2164–2179. 10.1038/nprot.2014.149 25122525

[B51] Kolesky D. B. Homan K. A. Skylar-Scott M. A. Lewis J. A. (2016). Three-dimensional bioprinting of thick vascularized tissues. Proc. Natl. Acad. Sci. U. S. A. 113 (12), 3179–3184. 10.1073/pnas.1521342113 26951646 PMC4812707

[B56] Lin Y. Wang K. Yan Y. Lin H. Peng B. Liu Z. (2013). Evaluation of the combinative application of SDS and sodium deoxycholate to the LC-MS-based shotgun analysis of membrane proteomes. J. Sep. Sci. 36 (18), 3026–3034. 10.1002/jssc.201300413 23832743

[B57] Ling T. Y. Liu Y. L. Huang Y. K. Gu S. Y. Chen H. K. Ho C. C. (2014). Differentiation of lung stem/progenitor cells into alveolar pneumocytes and induction of angiogenesis within a 3D gelatin--microbubble scaffold. Biomaterials 35 (22), 5660–5669. 10.1016/j.biomaterials.2014.03.074 24746968

[B58] Mahfouzi S. H. Amoabediny G. Safiabadi Tali S. H. (2021a). Advances in bioreactors for lung bioengineering: From scalable cell culture to tissue growth monitoring. Biotechnol. Bioeng. 118 (6), 2142–2167. 10.1002/bit.27728 33629350

[B59] Mahfouzi S. H. Tali S. H. S. Amoabediny G. (2021b). 3D bioprinting for lung and tracheal tissue engineering: Criteria, advances, challenges, and future directions. Bioprinting 21, e00124. 10.1016/j.bprint.2020.e00124

[B60] Mandrycky C. Wang Z. Kim K. Kim D. H. (2016). 3D bioprinting for engineering complex tissues. Biotechnol. Adv. 34 (4), 422–434. 10.1016/j.biotechadv.2015.12.011 26724184 PMC4879088

[B62] Matai I. Kaur G. Seyedsalehi A. McClinton A. Laurencin C. T. (2020). Progress in 3D bioprinting technology for tissue/organ regenerative engineering. Biomaterials 226, 119536. 10.1016/j.biomaterials.2019.119536 31648135

[B63] Meezan E. Hjelle J. T. Brendel K. Carlson E. C. (1975). A simple, versatile, nondisruptive method for the isolation of morphologically and chemically pure basement membranes from several tissues. Life Sci. 17 (11), 1721–1732. 10.1016/0024-3205(75)90119-8 1207385

[B64] Mendibil U. Ruiz-Hernandez R. Retegi-Carrion S. Garcia-Urquia N. Olalde-Graells B. Abarrategi A. (2020). Tissue-specific decellularization methods: Rationale and strategies to achieve regenerative compounds. Int. J. Mol. Sci. 21 (15), 5447. 10.3390/ijms21155447 PMC7432490 32751654

[B66] Mohgan R. Candasamy M. Mayuren J. Singh S. K. Gupta G. Dua K. (2022). Emerging paradigms in bioengineering the lungs. Bioeng. (Basel) 9 (5), 195. 10.3390/bioengineering9050195 PMC9137616 35621473

[B67] Murphy S. V. Atala A. (2014). 3D bioprinting of tissues and organs. Nat. Biotechnol. 32 (8), 773–785. 10.1038/nbt.2958 25093879

[B68] Nguyen-Truong M. Li Y. V. Wang Z. (2020). Mechanical considerations of electrospun scaffolds for myocardial tissue and regenerative engineering. Bioeng. (Basel, Switz. 7 (4), 122. 10.3390/bioengineering7040122 PMC7711753 33022929

[B69] Nichols J. E. Niles J. Riddle M. Vargas G. Schilagard T. Ma L. (2013). Production and assessment of decellularized pig and human lung scaffolds. Tissue Eng. Part A 19 (17-18), 2045–2062. 10.1089/ten.TEA.2012.0250 23638920 PMC3725800

[B70] Nikolova M. P. Chavali M. S. (2019). Recent advances in biomaterials for 3D scaffolds: A review. Bioact. Mater. 4, 271–292. 10.1016/j.bioactmat.2019.10.005 31709311 PMC6829098

[B71] Obata T. Tsuchiya T. Akita S. Kawahara T. Matsumoto K. Miyazaki T. (2019). Utilization of natural detergent potassium laurate for decellularization in lung bioengineering. Tissue Eng. Part C. Methods 25 (8), 459–471. 10.1089/ten.TEC.2019.0016 31317819

[B72] Owida H. A. Al-Nabulsi J. I. Alnaimat F. Al-Ayyad M. Turab N. M. Al Sharah A. (2022). Recent applications of electrospun nanofibrous scaffold in tissue engineering. Appl. Bionics Biomech. 2022, 1953861. 10.1155/2022/1953861 35186119 PMC8849965

[B73] Ozbolat I. T. Yu Y. (2013). Bioprinting toward organ fabrication: Challenges and future trends. IEEE Trans. Biomed. Eng. 60 (3), 691–699. 10.1109/TBME.2013.2243912 23372076

[B75] Persano L. Camposeo A. Tekmen C. Pisignano D. (2013). Industrial upscaling of electrospinning and applications of polymer nanofibers: A review. Macromol. Mat. Eng. 298, 504–520. 10.1002/mame.201200290

[B76] Prakash Y. S. Tschumperlin D. J. Stenmark K. R. (2015). Coming to terms with tissue engineering and regenerative medicine in the lung. Am. J. Physiology-Lung Cell. Mol. Physiology 309 (7), L625–L638. 10.1152/ajplung.00204.2015 PMC4593835 26254424

[B77] Rabbani M. Zakian N. Alimoradi N. (2021). Contribution of physical methods in decellularization of animal tissues. J. Med. Signals Sens. 11 (1), 1–11. 10.4103/jmss.JMSS_2_20 34026585 PMC8043117

[B78] Reing J. E. Brown B. N. Daly K. A. Freund J. M. Gilbert T. W. Hsiong S. X. (2010). The effects of processing methods upon mechanical and biologic properties of porcine dermal extracellular matrix scaffolds. Biomaterials 31 (33), 8626–8633. 10.1016/j.biomaterials.2010.07.083 20728934 PMC2956268

[B79] Ribitsch I. Baptista P. M. Lange-Consiglio A. Melotti L. Patruno M. Jenner F. (2020). Large animal models in regenerative medicine and tissue engineering: To do or not to do. Front. Bioeng. Biotechnol. 8, 972. 10.3389/fbioe.2020.00972 32903631 PMC7438731

[B80] Rider P. Kačarević Ž. P. Alkildani S. Retnasingh S. Barbeck M. (2018). Bioprinting of tissue engineering scaffolds. J. Tissue Eng. 9, 204173141880209. 10.1177/2041731418802090 PMC6176532 30305886

[B83] Scarritt M. E. Pashos N. C. Bunnell B. A. (2015). A review of cellularization strategies for tissue engineering of whole organs. Front. Bioeng. Biotechnol. 3, 43. 10.3389/fbioe.2015.00043 25870857 PMC4378188

[B84] Shojaie S. Ermini L. Ackerley C. Wang J. Chin S. Yeganeh B. (2015). Acellular lung scaffolds direct differentiation of endoderm to functional airway epithelial cells: Requirement of matrix-bound HS proteoglycans. Stem Cell. Rep. 4 (3), 419–430. 10.1016/j.stemcr.2015.01.004 PMC4375883 25660407

[B85] Singh D. Zo S. M. Kumar A. Han S. S. (2013). Engineering three-dimensional macroporous hydroxyethyl methacrylate-alginate-gelatin cryogel for growth and proliferation of lung epithelial cells. J. biomaterials Sci. Polym. Ed. 24 (11), 1343–1359. 10.1080/09205063.2012.759505 23796035

[B86] Skolasinski S. Panoskaltsis-Mortari A. (2018). Decellularization of intact lung tissue through vasculature and airways using negative and positive pressure. Methods Mol. Biol. 1577, 307–315. 10.1007/7651_2017_32 28656583 PMC5895527

[B87] Suki B. Bates J. H. (2008). Extracellular matrix mechanics in lung parenchymal diseases. Respir. physiology Neurobiol. 163 (1-3), 33–43. 10.1016/j.resp.2008.03.015 PMC2666313 18485836

[B88] Talò G. D'Arrigo D. Lorenzi S. Moretti M. Lovati A. B. (2020). Independent, controllable stretch-perfusion bioreactor chambers to functionalize cell-seeded decellularized tendons. Ann. Biomed. Eng. 48 (3), 1112–1126. 10.1007/s10439-019-02257-6 30963381 PMC7015956

[B90] Taylor D. A. Caplan A. L. Macchiarini P. (2014). Ethics of bioengineering organs and tissues. Expert Opin. Biol. Ther. 14, 879–882. 10.1517/14712598.2014.915308 24792885

[B91] Tebyanian H. Karami A. Nourani M. R. Motavallian E. Barkhordari A. Yazdanian M. (2019). Lung tissue engineering: An update. J. Cell. Physiol. 234 (11), 19256–19270. 10.1002/jcp.28558 30972749

[B92] Tsuchiya T. Sivarapatna A. Rocco K. Nanashima A. Nagayasu T. Niklason L. E. (2014). Future prospects for tissue engineered lung transplantation: Decellularization and recellularization-based whole lung regeneration. Organogenesis 10 (2), 196–207. 10.4161/org.27846 24488093 PMC4154954

[B93] Uhl F. E. Zhang F. Pouliot R. A. Uriarte J. J. Rolandsson Enes S. Han X. (2020). Functional role of glycosaminoglycans in decellularized lung extracellular matrix. Acta biomater. 102, 231–246. 10.1016/j.actbio.2019.11.029 31751810 PMC8713186

[B95] Wagner D. E. Bonvillain R. W. Jensen T. Girard E. D. Bunnell B. A. Finck C. M. (2013). Can stem cells be used to generate new lungs? *ex vivo* lung bioengineering with decellularized whole lung scaffolds. Respirol. Carlt. Vic.) 18 (6), 895–911. 10.1111/resp.12102 PMC3729745 23614471

[B96] Wallis J. M. Borg Z. D. Daly A. B. Deng B. Ballif B. A. Allen G. B. (2012). Comparative assessment of detergent-based protocols for mouse lung de-cellularization and re-cellularization. Tissue Eng. Part C. Methods 18 (6), 420–432. 10.1089/ten.tec.2011.0567 22165818 PMC3358122

[B97] Wanczyk H. Jensen T. Weiss D. J. Finck C. (2021). Advanced single-cell technologies to guide the development of bioengineered lungs. Am. J. Physiology-Lung Cell. Mol. Physiology 320 (6), L1101–L1117. 10.1152/ajplung.00089.2021 33851545

[B98] Weibel E. R. (2015). On the tricks alveolar epithelial cells play to make a good lung. Am. J. Respir. Crit. Care Med. 191 (5), 504–513. 10.1164/rccm.201409-1663OE 25723823

[B99] Weiss D. J. Bates J. H. Gilbert T. Liles W. C. Lutzko C. Rajagopal J. (2013). Stem cells and cell therapies in lung biology and diseases: Conference report. Ann. Am. Thorac. Soc. 10 (5), S25–S44. 10.1513/AnnalsATS.201304-089AW 23869447 PMC5475419

[B100] Wilkinson D. C. Alva-Ornelas J. A. Sucre J. M. Vijayaraj P. Durra A. Richardson W. (2017). Development of a three-dimensional bioengineering technology to generate lung tissue for personalized disease modeling. Stem Cells Transl. Med. 6 (2), 622–633. 10.5966/sctm.2016-0192 28191779 PMC5442826

[B101] Xie Z. Gao M. Lobo A. O. Webster T. J. (2020). 3D bioprinting in tissue engineering for medical applications: The classic and the hybrid. Polymers 12 (8), 1717. 10.3390/polym12081717 PMC7464247 32751797

[B102] Young B. M. (2019). Engineering the alveolar gas exchange barrier with extracellular matrix coatings for bioengineered lungs. Doctoral Dissertation (Virginia: Virginia Commonwealth University, Department of Biomedical Engineering).

[B103] Young B. M. Shankar K. Allen B. P. Pouliot R. A. Schneck M. B. Mikhaiel N. S. (2017). Electrospun decellularized lung matrix scaffold for airway smooth muscle culture. ACS Biomater. Sci. Eng. 3 (12), 3480–3492. 10.1021/acsbiomaterials.7b00384 33445384

[B104] Young B. M. Shankar K. Tho C. K. Pellegrino A. R. Heise R. L. (2019). Laminin-driven Epac/Rap1 regulation of epithelial barriers on decellularized matrix. Acta biomater. 100, 223–234. 10.1016/j.actbio.2019.10.009 31593773 PMC6892605

[B105] Yuan Y. Engler A. J. Raredon M. S. Le A. Baevova P. Yoder M. C. (2019). Epac agonist improves barrier function in iPSC-derived endothelial colony forming cells for whole organ tissue engineering. Biomaterials 200, 25–34. 10.1016/j.biomaterials.2019.02.005 30754017

[B106] Zhu W. Qu X. Zhu J. Ma X. Patel S. Liu J. (2017). Direct 3D bioprinting of prevascularized tissue constructs with complex microarchitecture. Biomaterials 124, 106–115. 10.1016/j.biomaterials.2017.01.042 28192772 PMC5330288

